# Remarks on the Use of Bromide of Iron

**Published:** 1851-03

**Authors:** Edward Gillespie

**Affiliations:** Brady’s Bend, Pa.


*Remarks on the use of Bromide of Iron.* By Edward Gillespie, M. D., of Brady's Bend, Pa.

Having been practising medicine for some five years past in the the neighborhood of Freeport, in Armstrong county, in this State, where a considerable quantity of Bromine is manufactured, I have been led to a somewhat extensive employment of a ferruginous compound of this article, the results of which have proved so satisfactory, that they may perhaps be not destitute of interest to the readers of the Examiner. The compound which I employ is a bromide of iron of my own preparation, and this is the only form in which I use the bromine. It has now in my hands entirely taken the place of iodine, appearing to me to meet all the indications for which that medicine has been exhibited, and with much greater efficiency. Indeed, the bromide has become one of my *indispensables* in practice, hardly subordinate in importance to my calomel or opium.

The cases in which I have been in the habit of using it are scrofulous tumours, inflammations of the glands, both acute and chronic, erysipelas, suppression of the menses, tetter, and in most cases where chalybeates are administered. Enlargement of the parotid, submaxillary, or cervical glands, whether from scrofula or other causes, I have never yet failed to discuss, provided they had not progressed to suppuration; and I may speak from an experience of not a few cases, and many of them of long standing. When suppuration does take place, the discharge soon ceases after lancing, and the orifice quickly heals by applying a solution of the bromide over the surface occupied by the tumor, and keeping a small plaster of basilicon ointment on the orifice. My method of using it in cases of tumors is to apply a small portion immediately over the tumor twice a day by means of a feather, and at the same time to administer it internally, commencing with eight or ten drops morning and evening in half a teacup of cold water, increasing a drop or two every day until nausea is produced, (which may be termed the point of toleration.) The dose is then to be reduced five or six drops and continued. This is the invariable rule I lay down for its internal use. I have given it to advantage in cases of felon, where a red and very painful streak would run up the arm, causing inflammation of the axillary glands with general febrile symptoms. Some of these cases present rather an alarming appearance for a time, but have always terminated favorably in my hands by a strict antiphlogistic treatment, and the use of the solution as a local application to the inflamed arm and glands.

Last spring an erysipelatous epidemic raged in a district of the country north-west of this, proving extremely fatal, and causing considerable alarm in the section where it prevailed. It exhibited quite a preference for the fair sex, nine-tenths of those attacked having been females. The first symptoms were chills, followed by a high grade of fever and sore throat; from 24 to 36 hours after the attack, the throat became so much swollen that deglutition was very painful, and in many cases entirely impeded. The breathing was very laborious. The tongue was also much swollen, so much so as in many cases to protrude from the mouth with an extremely thick black coat on the upper surface. In this stage it was generally called "black tongue." In from 48 to 72 hours from the commencement of the attack, when it pursued its natural course, the ears became red and inflamed, the redness and inflammation extending rapidly over the face, head, neck and shoulders. The eyes became entirely closed, the nose nearly covered by the swollen cheeks, the lips puffed out to an enormous extent, and the head, to use the expression then common, "as big as a bushel." Perhaps an idea may be conveyed of their appearance by likening it to that of persons drowned in warm weather, whose bodies have remained in the water a week or two, with the face and head painted scarlet after being removed. When they arrived at this point they were vulgarly termed "swell heads."

Occasionally the disease would appear on one ear and extend over the face to the median line. When this was the case, one side of the face would be swollen and the other perfectly natural, which gave the a patient very grotesque appearance. It would not remain long in this situation, however, until the sound ear would become red and the disease would soon hasten across the face to meet its companion.

When it did not appear on the skin or could not be brought out by artificial means, the patient generally died in a short time from suffocation. But it could in nearly all cases be brought out by the use of some highly stimulating lotion or the application of a fly blister to the neck. Still the danger was imminent either from sloughing and gangrenous inflammation of the cellular tissue, or a transfer of the disease to the brain. The patient, by external revulsion, was generally enabled to swallow, which of course was a strong point gained, and by the judicious use of internal remedies and proper local applications he now generally recovered. I must not omit to state that I found the free use of the lancet eminently serviceable whenever the state of the pulse required it.

With regard to local applications, I may say that I experimented with a variety of articles. In one case I applied exclusively the acetas plumbi in solution—in another the ethereal tincture of iodine, in another strong mercurial ointment, in another solution of corrosive sublimate, and finally the bromide of iron in solution. The progress of these different applications I watched for a couple of days, the internal treatment being the same in all. The acetate of lead appeared to have no influence on the disease whatever. It progressed as rapidly as though nothing were applied, although the solution was as strong as an ounce to a pint and a half of water, and kept constantly applied by means of muslin dipped in the solution.

The tincture of iodine appeared to do better—it retarded but did not arrest the disease. It was also strong, half an ounce to a pint of ether. The blue ointment I had tried previously in sporadic cases with a certain degree of success, but in this case it appeared to act but feebly.

The solution of corrosive sublimate had no other effect, than to produce violent salivation in fouror five days after its application.

The bromide of iron entirely arrested the disease in forty hours from its first application. I used it in all cases afterwards very successfully, by applying it two or three times per day to the parts affected, and extending it one or two inches over the sound skin, and the constant application of cloths wrung out of a solution of acetate of lead.

I have no very accurate formula for the solution of bromide of iron that I make use of. I put three ounces of bromine into ten ounces of water, then add four penny nails, which are about enough to take up all the bromine. If any are left, after the bromine is all taken up, I take them out, and add free bromine as long as it will be absorbed; if too much is added, I put in a nail or two to take up the surplus. It makes a dark liquid of an acid and styptic taste, which turns brick red when applied to the skin. This is the solution which I use.

[The bromide of iron (which is a hydrobromate of the protoxide) is liable, like the iodide, to partial decomposition from the oxygen of the atmosphere, free bromine being generated, and the sesquioxide of iron deposited. It should, therefore, for internal exhibition, be protected by saccharine matter. Mr. Dillwyn Parrish, of this city, has made, at our suggestion, a solution of the salt, with sugar, after the subjoined formula:—"Take of Bromine *two hundred grains ;* Iron Filings, *eighty-five grains ;* Distilled Water, *four and a half fluid ounces ;* Sugar, *three ounces.* Mix properly. Dose, ten minims, to be repeated and gradually increased, ten minims containing a grain of the bromide."—Eds. Ex.]

